# Application of Multidomain Cognitive Training in a Tele-Neurorehabilitation Setting for Treatment of Post-Stroke Cognitive Disorders

**DOI:** 10.3390/brainsci15010011

**Published:** 2024-12-26

**Authors:** Marianna Contrada, Loris Pignolo, Martina Vatrano, Caterina Pucci, Isabel Mantia, Federica Scarfone, Maria Quintieri, Antonio Cerasa, Gennarina Arabia

**Affiliations:** 1 S.Anna Institute, 88900 Crotone, Italy; mariannacontrada@gmail.com (M.C.); martinavatrano92@gmail.com (M.V.); c.pucci@isakr.it (C.P.); isabel.mantia@virgilio.it (I.M.); federica.scarfone10@gmail.com (F.S.); m.quintieri@isakr.it (M.Q.); antonio.cerasa@cnr.it (A.C.); 2 IBSBC-CNR, Via T. Campanella, 88100 Catanzaro, Italy; 3 Institute of Neurology, University Magna Graecia, 88100 Catanzaro, Italy; g.arabia@unicz.it

**Keywords:** Tele-NeuroRehabilitation, multidomain cognitive training, stroke, memory, language, mood

## Abstract

Purpose: Cognitive dysfunctions are still very common in the chronic phase of stroke when patients are discharged from neurorehabilitation centers. Even individuals who appear to have made a full clinical recovery may exhibit new deficiencies at home. Here, we present evidence of a novel kind of therapy at home aimed at contrasting the heterogenic evolution of stroke patients using a multidomain cognitive approach. Methods: Eighteen ischemic stroke patients were assessed in a within-subject longitudinal design (age 62.33 ± 11.1 years; eight men). Patients underwent the Tele-NeuroRehabilitation (TNR) multidomain cognitive training treatment using the Virtual Reality Rehabilitation System (VRRS) five times a week for 1 h sessions for four consecutive weeks. The protocol included the stimulation of specific cognitive functions, such as logical skills, praxis skills, attention, executive functions, memory, space time orientation and perception, and speech therapy. To determine neuropsychological changes, patients were evaluated before the sessions (T0), at the end of the sessions (T1), and after six months (T2). Results: The multidomain cognitive training induced a significant improvement in the working memory and language abilities as well as depression symptoms and alleviated caregiver burden. Most of this cognitive enhancement persisted after six months (T2), with the exception of depression symptoms. Otherwise, a significant decline in attention abilities was reported, thus demonstrating a lack of effect in this function. Conclusions: Our results suggest that multidomain cognitive TNR is a suitable protocol for reducing some cognitive and behavioral alterations in patients with strokes, with a beneficial impact also on the caregivers’ burden distress management. Further RCTs are warranted to validate this new kind of approach.

## 1. Introduction

A total of 9.4 million American people, or roughly 3.6% of the country’s adult population, report having experienced a stroke, according to the American Heart Association’s 2023 Statistical Update. After a stroke, cognitive impairment might appear years later or sooner [1]. Depending on the area of the brain affected and the degree of the lesions, strokes can result in long-lasting functional and cognitive deficits that cause severe and prolonged disability, significantly reduce the quality of life for patients [2,3], and increase the burden on caregivers [3,4]. Up to 60% of individuals who have had an ischemic stroke experience cognitive impairment with incidence rates ranging from 20% to 80% [5]. However, the results of the research vary depending on nationality, race, and diagnostic standards. According to the MMSE, the prevalence of cognitive impairment three months following a stroke varies from 24% to 39% in European countries like Sweden and Britain [6,7]. These people could be classified as having moderate cognitive impairment or dementia based on the severity of their cognitive deterioration. It is interesting to note that the dementia ratio within three months following a stroke ranges from 6% to 27% in various studies [8,9]. Cognitive impairments after stroke include deficits in memory process, attention, executive functions, and visuo-spatial ability. These deficits are also frequent in patients with apparently successful clinical recovery and no functional motor disability [2,10,11]. Follow-up studies show that cognitive dysfunctions are quite persistent in stroke patients and remain highly prevalent in the following month, and up to three years after the acute event [12,13]. After discharge from the hospital rehabilitation unit, it is difficult for patients to receive appropriate care, with consequent functional decline that can affect the long-term outcome of stroke [14].

One innovative method for treating cognitive deficits after discharge from the hospital is Tele-NeuroRehabilitation (TNR). Telerehabilitation overcomes obstacles like geographic distance and restricted access to specialized treatment by utilizing digital platforms to guarantee constant and customized therapy [15]. Its effectiveness in enhancing cognitive areas such as memory, attention, and executive function through personalized, structured, and interactive activities has been demonstrated by several studies [16]. Additionally, telerehabilitation increases patient adherence and interest while offering a channel for ongoing observation and feedback [17,18]. This technique not only enhances rehabilitation outcomes but also satisfies the growing need for conveniently accessible, cost-effective, and scalable healthcare options for stroke survivors. There is evidence from some systematic reviews that TNR improves neurological patients’ cognitive functioning just as much as traditional face-to-face therapy [19,20].

The impact of TNR-related cognitive therapies on specific areas of cognitive function following a stroke, including memory, executive function, attention, apraxia, neglect, and perception, has been examined in Cochrane studies [17]. Despite TNR interventions having been demonstrated to be effective, some authors pointed out that improvements did not enhance stroke patients’ function and were unlikely to last over time [21]. Generally, objective post-stroke cognitive recovery ranged from 15% to 30% after one year from the event [22,23]. A recent meta-analysis [24] demonstrated that executive function was the domain that most strongly correlated with overall cognitive recovery from intervention. This was followed by memory, consciousness, visuoperceptual, and psychomotor speed functions. Global cognition, attention, language, and orientation are only partially recovered. Because post-stroke cognitive impairment is complex and frequently widespread, concentrating on domain-specific cognitive outcomes may not adequately capture the interrelated cognitive abnormalities that occur in stroke [25]. The limited scope of certain cognitive rehabilitation strategies that concentrate on a single domain of a cognitive function still must be addressed. Nevertheless, the heterogeneity of cognitive interventions that could be provided by TNR systems could compromise the validity and surely hinder the generalization of the results to different contexts.

For this reason, in this pilot longitudinal study, we sought to evaluate the effectiveness of a multidomain cognitive training delivered by a well-validated TNR system on stroke patients (Virtual Reality Rehabilitation System, VRRS of the Khymeia group, Noventa Padovana, Italy; https://khymeia.com/it/, accessed on 10 October 2024).

## 2. Material and Methods

### 2.1. Participants

From January 2022 to June 2024, all patients who received discharge from the intense rehabilitation unit of the Institute S. Anna in Crotone were consecutively assessed to determine which participants met the study’s inclusion and exclusion requirements. Inclusion criteria were (a) patients who have been diagnosed with ischemic stroke, where the type of stroke could have been ischemic in the territories of the middle or anterior cerebral artery; (b) >18 years; (c) presence of mild motor impairment and persistence of cognitive deficits; (d) stable clinical condition; (e) time from event ≥ 8 months; (f) absence of complications (i.e., infections, seizures); and (g) availability of receiving in-home NeuroRehabilitation service. We excluded patients with (a) presence of other non-vascular brain lesions; (b) history of dementia and psychiatric disorders; (c) history of regular prior and/or current drug and/or alcohol abuse; (d) cognitive impairment (Mini Mental State Examination (MMSE) score > 24); (e) aphasia, as assessed by the Aachener Aphasie Test (AAT); and (f) severe visual deficits.

All participants gave written informed consent. The study was approved by the Ethical Committee of the Central Area Regione Calabria (n. 113; 17 April 2018), according to the Helsinki Declaration.

### 2.2. Study Design

**Figure 1:**
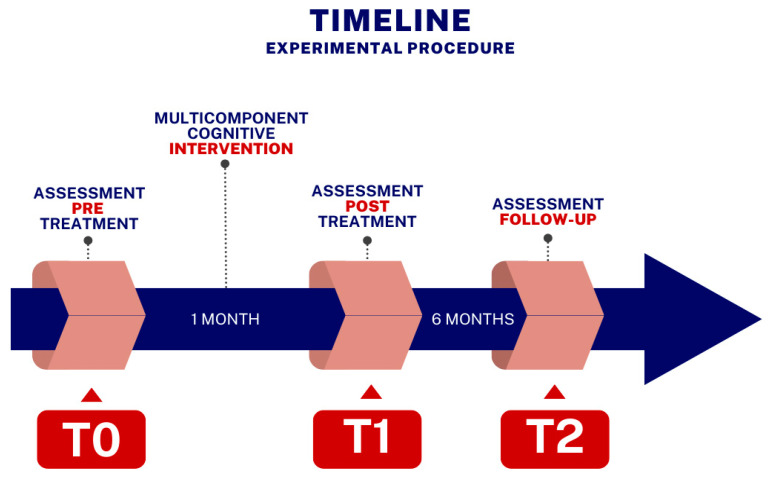
Timeline of experimental procedure. T0: baseline; T1: 4 weeks post-treatment; T2: 6 months post-treatment.

We employed a within-subject design with four main phases (Figure 1). The initial phase was centered on patient recruitment (see to the inclusion criteria mentioned above). Data entry assistants and neuropsychologists were blinded to every stage of the investigation. Stroke patients were enrolled for TNR treatment after discharge from the Intensive Rehabilitation Unit. The eligible stroke patients were evaluated cognitively at baseline (T0) in the second stage. In the third stage, individuals received multidomain cognitive TNR training where subjects received home-based VRRS of the Khymeia group, with treatment lasting for 1 h/day, 5 days/week, for 4 weeks (60 min sessions of individualized cognitive rehabilitation). Lastly, utilizing the same technique as at baseline (T0), participants received a blinded new cognitive and clinical assessment from a neuropsychologist at the end of the 4-week training session (T1) and 6 months later (T2). 

### 2.3. Clinical and Neuropsychological Assessment

All participants were tested at every timepoint, first, using the Mini-Mental State Examination (MMSE) [26] for assessing general cognitive functioning, and then, using the Cognitive Reserve Index questionnaire (CRIq) [27].

Next, we evaluated single cognitive functions using (a) Rey Auditory Verbal Learning Test (RAVLT) [28], for assessing verbal learning and memory, including proactive inhibition, retroactive inhibition, retention, and differentiating encoding versus retrieval difficulties; (b) Digit Span (Verbal and Spatial Immediate Memory Span) [29] to assess short-term memory through the auditory presentation of a series of items of increasing length to be repeated in the same order of presentation (direct span, Forward, FW) or in reverse (reverse span, Backward, BW); (c) Trail Making Test A-B (TMT A-B) [30] to assess sustained attention, task switching, and spatial planning ability in a visuomotor task; (d) copying of drawings test without programming elements (CD) and copying of drawings test with programming elements (CDP) [28] to assess praxic skills in copying geometric drawings with programming elements and without programming elements; and (e) action and object naming subtests from the battery for the assessment of aphasic disorders (Battery for Analysis of Aphasics Deficit, B.A.D.A.) [31] for language production.

Finally, the following questionnaires were used for mood assessment: (a) Beck Depression Inventory II (BDI-II) [32]; (b) State-Trait Anxiety Inventory (STAI) [33] to assess the presence of state anxiety (X-1) and trait anxiety (X-2); and (c) Caregiver Burden Inventory (CBI) [34]. The Short Form Health Survey-36 (SF-36) [35] questionnaire was administered to assess quality of life and mental component summary (MCS) and physical component summary (PCS).

### 2.4. Cognitive VRRS Treatment

The VRRS HomeKit (Khymeia group, Padova, Italy) was used for the TNR training. The VRRS-related modules offer a wide variety of cognitive exercises clinically validated and specifically designed to train attention, memory, visual-spatial ability, praxia, language, and speech that could be compromised in post-stroke patients or lost during the chronic phase at home. With sensors like a K-wand and K-sensors, the device is a tablet that fits in a carrying bag and enables a whole training program, including motor, cognitive, and speech therapy modules, to be conducted at home. The therapist (a neuropsychologist or physiotherapist) uses the teleworkstation (also known as the Tele-Cockpit) to support the patient during each training session, and the caregiver acts as a co-therapist to help the therapist. The system includes teletraining, telemonitoring, teleconsultation, and streaming of diagnostic imaging.

TNR-VRRS sessions included the stimulation of specific cognitive domains:

Logical skills
Logical-mathematical skills
Praxis skills
Attention
Executive functions
Memory
Space-time orientation
Spatial perception
Speech therapy

The TNR-VRRS technology allows the creation of exercise programs highly customized to the patient’s cognitive abilities. Each exercise can be regulated in terms of duration, number of repetitions, initial difficulty level, and number of repeats per level. The device provides audible feedback to encourage the patient and explain to them their performance. Every patient completed the activities in the same sequence, three for each function (see Table 1).

### 2.5. Statistical Analysis

Statistical analysis was performed using SPSS 26 software (version 26; Statistical Package for Social Sciences; www.spss.it), and graphs were generated using JMP software (version 16, SAS Institute, Cary, NC, USA). Summary statistics are expressed as means and standard deviations. The non-parametric exact test was used for the statistical analysis. The neuropsychological and mood assessments were compared across timepoints for each group by the Wilcoxon test. The level of significance was set at *p* < 0.05. The effect size was calculated as the absolute value of Z/√(N) for the Wilcoxon test, where Z is the Z-statistic of the statistical test, and N is the total number of subjects. The effect size results were considered: r < 0.1, not significant; 0.1 ≤ r < 0.3, low; 0.3 ≤ r < 0.5, medium; r ≥ 0.5, high.

## 3. Results

**Figure 2:**
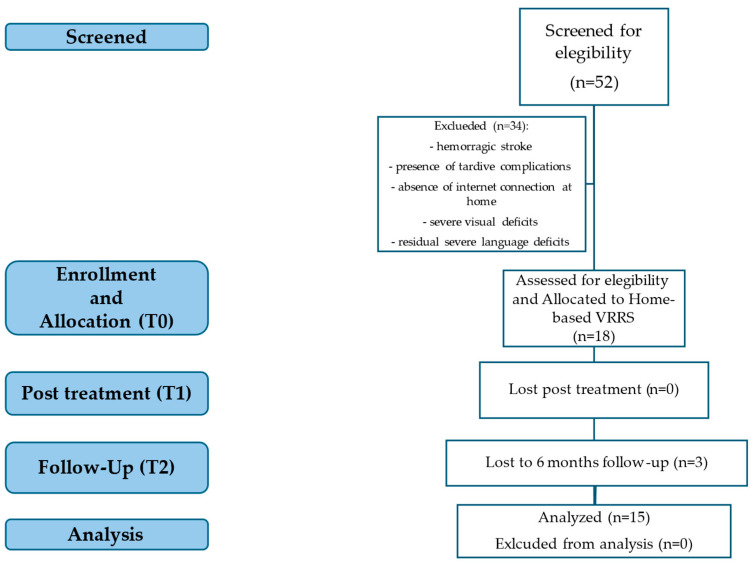
Flow diagram showing subject recruitment, assignment, and analysis procedures.

From an initial sample of 52 ischemic stroke patients, n = 34 were excluded because they did not meet the study inclusion criteria. Eighteen fully met the admission criteria and were enrolled in the present study (Figure 2). Three patients did not terminate the T2 at 6 months after treatment. One patient died, and two patients stopped because of medical complications. All patients took conventional drug therapy including anticoagulants and antiplatelets. Every patient thought the TNR intervention was easy to use and intuitive. The patients’ characteristics are reported in Table 2.

**Figure 3:**
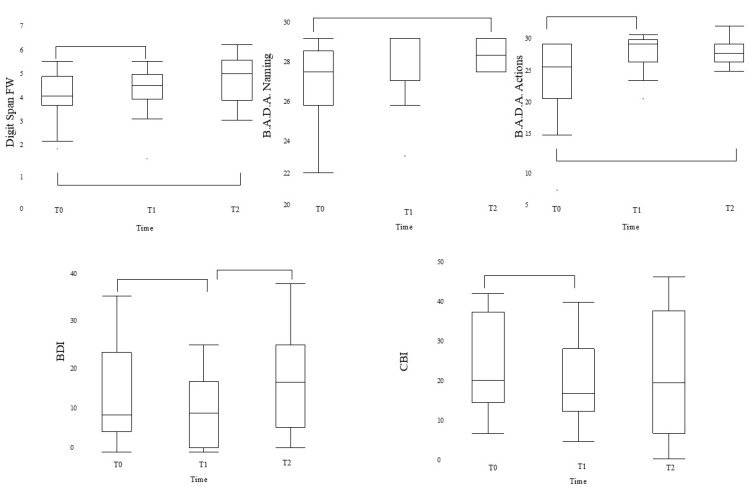
Plots of most relevant cognitive (**above**) and behavioral (**down**) changes after TNR.

After treatment, we detected significant improvements between T0 and T1 timepoints in digit span FW (Z = −1.78, *p* = 0.04, r = 0.39) and B.A.D.A. actions (Z = −2.94, *p* < 0.0001, r = 0.69), where performance also tended to be improved after 6 months (Z = −2.35, *p* = 0.008, r = 0.61; Z = −1.94, *p* = 0.03, r = 0.50; Z = −2.41, *p* = 0.008, r = 0.62; respectively; for digit span FW and B.A.D.A. actions and naming) (Table 3, Figure 3).

The mood evaluation revealed decreasing values in the BDI II between T0 and T1 (Z = −2.33, *p* = 0.008, r = 0.55) and between T1 and T2 (Z = −2.194; *p*-level = 0.01,r = 0.52), while CBI resulted in being improved at T1 (Z = -1.97, *p* = 0.026, r = 0.46), remaining stable after 6 months (Table 3, Figure 3).

On the other hand, performance in the attention domain worsened between T0 and T1 in the TMT B (Z = −1.92, *p* = 0.03, r = 0.45) and TMT B-A (Z = −2.33, *p* = 0.009, r = 0.55), and between T1 and T2 (Z = −1.88, *p* = 0.03, r = 0.44; Z = −1.80, *p* = 0.04, r= 0.46; respectively; for TMT B and TMT B-A) but remained stable at T2 follow-up evaluation (Table 3). Similar analyses have been conducted for MCS and PCS, but no statistically significant differences were found.

Finally, to evaluate the effect of gender, the entire sample was divided into two groups: male (eight subjects, age 62.33 ± 11.16) and female (10 subjects, age 63.20 ± 11.58). In the female group, we detected significant improvements between the T0 and T1 timepoints in B.A.D.A. actions (Z = −2.37, *p* = 0.008, r = 0.75), where performance also tended to be improved after 6 months with respect to the male group (Z = −2.17, *p* = 0.016, r = 0.69; Z= −2.17, *p* = 0.016; r = 0.69; Z = −1.83, *p* = 0.04, r = 0.58; respectively; for digit span FW and B.A.D.A. naming and actions). The mood evaluation revealed decreasing values in the BDI II between T0 and T1 (Z = −2.35, *p* = 0.008, r = 0.74), and between T1 and T2 (Z = −2.31; *p*-level = 0.01, r = 0.73), while, for the male group, only CBI was found to be improved at T1 (Z = −2.02; *p*-level = 0.03, r = 0.64). Similar analyses were conducted by dividing the group by age, but no statistically significant differences were found.

## 4. Discussion

The aim of this study is to demonstrate the effectiveness of multidomain cognitive training in chronic stroke patients at home exploiting the potential of telerehabilitation devices. Post-stroke cognitive impairment is generally diagnosed about three to six months after the cerebrovascular event, by which time the brain would have recovered at least partially from the event [36]. Therefore, the distance to the event could be an indicator of the effectiveness of Tele-NeuroRehabilitation on cognitive domains.

Short-term post-hospital discharge telerehabilitation programs have generally been shown to be successful in enhancing certain cognitive abilities, such as language and memory [16,20], but there is little evidence of their effect on behavioral symptoms [17]. With respect to previous telerehabilitation applications, now we proposed a wider cognitive intervention for stimulating all cognitive functions. Despite the within-subject approach used in this study, we demonstrated that the TNR multidomain cognitive approach can induce significant improvement in working memory (digit span FW), language (B.A.D.A. actions/naming), depression, and caregiver burden, with improvement also persisting in almost all these domains six months after therapy. Otherwise, the proposed cognitive treatment failed to contrast cognitive decline in attentional functions.

Generally, traditional cognitive rehabilitation and telerehabilitation approaches in stroke patients have often been focused on a single domain approach [16]. Instead, multidomain cognitive training programs involve many activities that interact and put more strain on the cognitive system rather than just one domain (such memory or processing speed) [37]. As it has been shown in other clinical settings, people’s motivation to train can be increased by offering them interesting, varied programs with substantial enjoyment components in order to maximize their general cognitive performance [38]. Using a multi-component intervention (called MCI-SET, including exercises for cognitive formation, motor-cognitive skills, and cognitive resiliency) [39] demonstrated significant improvements of general cognition, short-term memory, attention, and function execution in older people. Similarly, Manenti et al. [40], using a similar multidomain cognitive approach (VRRS, Khymeia), found increased performance in memory, language, and visuo-constructional abilities compared to face-to-face traditional treatment in 49 mild cognitive impairment (MCI) patients. When patients continued to be treated at home with a multidomain cognitive TNR approach, authors described the maintenance of the obtained gains rather than home-based unstructured stimulation. Considering older people or those in preclinical phase of dementia, Nousia et al. [41] and Tagliabue et al. [42] confirmed the efficacy of the multidomain approaches in recovering memory, language, and executive functions. However, in our study, we detected a lack of effect in attention abilities, which contrasts with previous studies where the application of multidomain cognitive training was able to recover this function in patients with MCI [43] or in older people [38].

Finally, the evidence that our approach impacts significantly on depression symptomatology is in line with previous studies. Ng et al. [44] proposed a multidomain intervention including nutritional supplementation, physical training (N = 48), and cognitive training in older people, demonstrating improved depression scores measured with the Geriatric Depression Scale. Similar results were reported by Roh et al. [45], using another kind of multidomain intervention including physical activity, healthy diet, social activity, and brief cognitive restructuring in older adults diagnosed with major depressive disorder.

### Limitations

The first limitation is the absence of a control group to assess TNR’s effects in comparison to traditional therapies. It is crucial to remember that, despite this limitation, this study is aimed at providing preliminary evidence of the impact of this kind of treatment in chronic stroke patients at home. Next, the employment of a large multidomain cognitive training contrasts with the traditional single-domain approach where people are engaged in specific and longer sessions aimed at improving specific cognitive functions. A direct comparison of the two methods is warranted in future RCT studies.

## 5. Conclusions

The best evidence for cognitive rehabilitation, according to current trends in the research, occurs when patient-reported results regarding involvement in functional tasks are compared to measured performance in specific functions within the various cognitive domains [45]. Several studies have demonstrated the efficacy of telerehabilitation on cognitive abilities in individuals with stroke [16]. However, despite this evidence, there is no clinical agreement regarding the modality and content-design of telerehabilitation, and these therapies are provided in a variety of methods. Here, we provided preliminary evidence that a multidomain cognitive approach might stimulate several cognitive and behavioral resources with also long-lasting effects in chronic stroke patients at home. We observed improvements in verbal short-term memory (digit span FW), a skill essential for learning new information, and language abilities (associated to recall of words concerning activities and behavior, BADA verbs). Even six months after therapy ends, these long-lasting gains are sustained.

Additionally, the patient’s long-term mood and the caregiver’s felt stress were both improved by the treatment. As a result, TR therapy has a far-reaching impact on the family environment, lowering the perceived stress of the caregiver in addition to being successful in curing the patient’s cognitive and psychological symptoms.

## Figures and Tables

**Table 1:** Multidomain cognitive VRRS modules.

Main Domain	Task	Description	Task Duration
Logical-mathematical skills	Logical associations—images/words	Images/words appear on the screen to be matched according to a logical relationship.	10’
	Find the extraneous word/image	The subject must find the unrelated word/image.	10’
	Calculate total price/rest	The subject must figure out how much needs to be paid in total or how much change is owed based on the information displayed on the screen.	10’
Spatial perception/praxis skills	Puzzle	To create an accurate and whole jigsaw puzzle, the subject must rearrange a collection of jumbled jigsaw pieces.	10’
	Drawing by neglect	An incomplete figure appears on the screen on one side to be completed by the patient.	10’
	Rotation	Objects with different rotations appear on the screen. To finish a sequence, the patient must identify which rotation is accurate	10’
Attention	Attentional matrices	A sheet with one or more matrices (stimulus/target) to be crossed in a grid with many distractions will show up on the screen.	10’
	Recognize/match banknotes/coins	An overview screen will be presented with a series of random banknotes or coins in disarray. The task will be to recognize or match the front or back banknotes or coins.	10’
	Find differences	The subject will have to find the differences between two apparently identical images.	10’
Executive functions	Planning	Snatches of a brief story are presented on screen in a random order. The participant must put them back in chronological order.	10’
	Change color/shape/dimension/all	The subject is asked to choose from a set of figures a geometric figure that differs from the target simply in terms of shape, only in terms of color, only in terms of size, or in terms of color, shape, and dimension altogether.	10’
	Collect money up	A set of coins (starting with cents) or a set of banknotes (starting with EUR 5) appear on the screen. The subject is asked to collect the indicated amount.	10’
Memory	Open safe (backward/forward)	A closed safe will appear on screen and a sequence of numbers to be memorized will be shown. After a few seconds, the numbers will disappear and to open the safe, the subject must put the sequence in the same order or backward.	10’
	Visual memory	On the screen, pairs of cards (geometric shapes or animals) will be presented for the person to memorize. Then, the cards will turn over and the person will have to remember the position of the pairs.	10’
	Word memorization	A list of words that shows up on the screen must be committed to memory by the user. These terms will then vanish and turn up in a list of distracting words.	10’
Language	Identify the action	The subject must identify the action illustrated on screen.	10’
	Reconstruct the word	Letters appear on screen that the participant must utilize to piece together the correct word.	10’
	Separate by semantic group	The task requires the subject to sort things into groups based on the semantic categories to which they belong.	10’

**Table 2:** Participant characteristics.

Subject	Gender	Age	Diagnosis	Time from Event (Days)	MMSE	CRIq Total
1	M	65	Left fronto-temporal stroke	734	24	83
2	M	69	Left fronto-temporal stroke	491	18.27	78
3	F	63	Left parieto-temporal stoke	420	22.27	68
4	M	62	Bilateral frontal stroke	1220	24	92
5	F	43	Left cerebellar stroke	488	24	110
6	F	67	Right insulo-temporo-parieto-frontal stroke	914	24	143
7	F	41	Right thalamic stroke	1038	24	77
8	M	49	Right occipito-parietal stroke	240	24	100
9	M	53	Right parietal stroke	511	24	84
10	M	68	Left temporal stroke	250	24	83
11	F	62	Right temporal fronto-parietal stroke	777	24	129
12	M	49	Tail stroke of the right ventricle nucleus	755	24	93
13	F	75	Left cerebellar stroke	368	24	91
14	F	50	Right temporal fronto-parietal stroke	384	23.2	93
15	F	73	Left pontine stroke	290	24	95
16	F	76	Right frontoparietal stroke	384	23.3	124
17	M	75	Right temporal fronto-parietal stroke	225	24	94
18	F	76	Right cerebellar stroke	269	24	127
Mean (SD)	8 M 10 F	62.33 (11.16)		542 (296.86)	23.57 (1.44)	98.00 (20.58)

Data are shown as mean and standard deviation. R (right); L (left); MMSE (Mini Mental State Examination); CRIq (Cognitive Reserve Index questionnaire).

**Table 3:** Timeline of neuropsychological assessments in the interventional TNR group.

	Timepoints			Post Hoc Comparisons		
	T0	T1	T2	T0 vs. T1 *Z*-/*p*-Values	T1 vs. T2 *Z*-/*p*-Values	T0 vs. T2 *Z*-/*p*-Values
MEMORY						
RAVLT	43.62 (11.69)	41.18 (9.31)	43.43 (12.41)	z = −0.88; *p* = 0.20	z = −1.07; *p* = 0.16	z = −0.39; *p* = 0.37
Digit Span FW	4.09 (1.19)	4.46 (1.20)	4.98 (1.16)	z = −1.78; *p* = 0.04	z = −1.36; *p* = 0.09	z = −2.35; *p* = 0.008
Digit Span BW	3.29 (1.52)	3.20 (1.26)	3.67 (0.84)	z = −0.35; *p* = 0.38	z = −0.53; *p* = 0.31	z = −1.63; *p* = 0.06
VISUO-SPATIAL ABILITIES						
CD	9.67 (2.38)	10.54 (2.06)	10.08 (1.25)	z = −1.21; *p* = 0.13	z = −0.36; *p* = 0.38	z = −1.68; *p* = 0.05
CDP	67.23 (5.19)	61.31 (18.00)	62.62 (8.88)	z = −1.00; *p* = 0.18	z = −0.56; *p* = 0.32	z = −0.94; *p* = 0.22
ATTENTIONAL/EXECUTIVE FUNCTIONS						
TMT A (s)	81.66 (57.47)	70.66 (53.04)	73.76 (50.32)	z = −1.35; *p* = 0.09	z = −0.20; *p* = 0.43	z = −1.33; *p* = 0.10
TMT B (s)	156.39 (77.2)	272.70 (251.2)	224.69 (125.7)	z = −1.92; *p* = 0.03	z = −1.88; *p* = 0.03	z = −1.07; *p* = 0.16
TMT B−A (s)	84.61 (61.54)	202.04 (200.4)	133.84 (113.8)	z = −2.33; *p* = 0.009	z = −1.80; *p* = 0.04	z = −0.87; *p* = 0.22
LANGUAGE						
B.A.D.A. Naming	27.39 (2.43)	28.12 (3.04)	29.09 (0.94)	z = −1.39; *p* = 0.09	z = − 0.17; *p* = 0.46	z = −2.41; *p* = 0.008
B.A.D.A. Actions	24.33 (4.41)	27.18 (2.00)	27.00 (1.55)	z = −2.94; p < 0.001	z = −1.27; *p* = 0.13	z = −1.94; *p* = 0.03
MOOD						
BDI-II	16.11 (11.74)	11.61 (8.49)	19.09 (11.61)	z = −2.33; *p* = 0.008	z = −2.19; *p* = 0.01	z = −0.18; *p* = 0.44
STAI XI	42.17 (9.78)	42.22 (10.58)	40.79 (6.91)	z = −0.63; *p* = 0.28	z = −0.67; *p* = 0.27	z = −0.09; *p* = 0.47
STAI XII	45.33 (7.45)	43.67 (12.42)	45.93 (11.98)	z = −0.85; *p* = 0.21	z = −1.11; *p* = 0.14	z = −0.25; *p* = 0.41
CBI	27 (10.95)	23.56 (9.65)	25.85 (14.83)	z = −1.97; *p* = 0.03	z = −0.09; *p* = 0.48	z = −0.87; *p* = 0.21
QUALITY OF LIFE						
SF-36 MCS	49.34 (16.19)	49.68 (19.19)	58.47 (23.56)	z = −0.53; *p* = 0.31	z = −1.33; *p* = 0.10	z = −1.10; *p* = 0.15
SF-36 PCS	41.81 (18.15)	43.05 (17.60)	49.32 (19.94)	z = −0.51; *p* = 0.32	z = −0.36; *p* = 0.37	z = −1.45; *p* = 0.08

Rey Auditory Verbal Learning Test (RAVLT); digit span (verbal and spatial immediate memory span); direct span, forward (FW) or in reverse span, backward (BW); copying of drawings test with programming elements (CDP); Trail Making Test A-B (TMT A-B); object naming and action naming (Battery for Analysis of Aphasics Deficit, B.A.D.A.); Beck Depression Inventory II (BDI-II); State-Trait Anxiety Inventory (STAI); Caregiver Burden Inventory (CBI); Short-Form 36 (SF-36); Mental Component Summary (MCS) and Physical Component Summary (PCS).

## Data Availability

The raw data supporting the conclusions of this article will be made available by the authors on request due to due to privacy and ethical restrictions.
